# Improving the Adaptability of Simulated Evolutionary Swarm Robots in Dynamically Changing Environments

**DOI:** 10.1371/journal.pone.0090695

**Published:** 2014-03-05

**Authors:** Yao Yao, Kathleen Marchal, Yves Van de Peer

**Affiliations:** 1 Department of Plant Systems Biology, VIB, Ghent, Belgium; 2 Department of Plant Biotechnology and Bioinformatics, Ghent University, Ghent, Belgium; 3 Department of Microbial and Molecular Systems, KU Leuven, Leuven, Belgium; 4 Department of Information Technology, iMinds, Ghent University, Ghent, Belgium; 5 Department of Genetics, Genomics Research Institute, University of Pretoria, Pretoria, South Africa; Mount Sinai School of Medicine, United States of America

## Abstract

One of the important challenges in the field of evolutionary robotics is the development of systems that can adapt to a changing environment. However, the ability to adapt to unknown and fluctuating environments is not straightforward. Here, we explore the adaptive potential of simulated swarm robots that contain a genomic encoding of a bio-inspired gene regulatory network (GRN). An artificial genome is combined with a flexible agent-based system, representing the activated part of the regulatory network that transduces environmental cues into phenotypic behaviour. Using an artificial life simulation framework that mimics a dynamically changing environment, we show that separating the static from the conditionally active part of the network contributes to a better adaptive behaviour. Furthermore, in contrast with most hitherto developed ANN-based systems that need to re-optimize their complete controller network from scratch each time they are subjected to novel conditions, our system uses its genome to store GRNs whose performance was optimized under a particular environmental condition for a sufficiently long time. When subjected to a new environment, the previous condition-specific GRN might become inactivated, but remains present. This ability to store ‘good behaviour’ and to disconnect it from the novel rewiring that is essential under a new condition allows faster re-adaptation if any of the previously observed environmental conditions is reencountered. As we show here, applying these evolutionary-based principles leads to accelerated and improved adaptive evolution in a non-stable environment.

## Introduction

An important goal in evolutionary robotics is the development of systems that show self-adaptation in dynamically changing environments [1], [2]. Searching for the ‘fittest phenotype’ is only one aspect of the self-adaptive behaviour of such so-called complex adaptive systems (CASs), because under a dynamically changing environment, a solution that is optimal at a certain time might be different from an optimal solution at a later time. A truly self-adaptive system thus should not only reach higher performance in one particular environment, but should also evolve a better self-innovating ability that allows it to survive under different and changing conditions. This requires the ability to learn from past experiences, because although environmental changes are unpredictable, they are likely to reoccur.

Being naturally occurring examples of complex adaptive systems, biological systems provide an important source of inspiration [3]–[6]. The molecular mechanisms underlying the adaptability of biological systems are Gene Regulatory Networks (GRNs), which are composed of interacting genetic entities such as genes and proteins [7]–[9]. These networks transduce signals rising from environmental cues into a proper phenotypic behaviour that allows the organism to flexibly respond to environmental changes. The signalling networks active in a cell are the result of an underlying genetic encoding, provided by the genome. Evolutionary processes acting on this genome gradually can lead to novel emerging circuits (evolutionary network rewiring).

Several bio-inspired systems have been developed that use an artificial genome (AG) and a corresponding controller, usually a network structure represented by an Artificial Neural Network (ANN) [10], [11]. Here, a distinction can be made between systems that rely on a direct versus an indirect encoding. Systems that make use of direct coding use an ANN network design with an a-priory defined structure that directly determines the robots' phenotype. Such systems are generally well suited to efficiently evolve an optimal behaviour towards a particular predefined task because they have very good learning abilities [10]. Systems that use indirect coding do not impose a predefined network structure, but only predefine rules. For instance, a ‘gene’ will define a node in the ANN, but this node will find and connect with other nodes in the ANN based on the given conditions. The ultimate structure of the network will therefore develop itself, according to the predefined rules. Compared to a system that makes use of a direct coding scheme, one that uses indirect coding in general allows for a more compact and flexible encoding of the genome and its corresponding GRN, mainly because not all details of the network structure need to be predefined and the GRN structure can evolve during the developmental process [12], [13]. Such indirect coding approach is therefore more suitable to develop self-adaptive systems. Recent indirect coding approaches encode their ‘rules’ with a more biologically realistic version of an AG that mimics features of real biological genomes, for instance by means of mimicking an encoding of transcriptional interactions between TFs and their targets [14]–[18].

However, most earlier implementations have in common that, irrespective of their structure and implementation specificities, the evaluation of fitness or performance acts directly on the network controller by either affecting its structure or the weights of its interactions whereas the AG serves as nothing more than a convenient encoding of the GRN on which the evolutionary algorithms are applied. In contrast with real biological systems, most of these previously developed approaches do not allow for an uncoupling between the genomic encoding and the part of the genome that is activated in a condition-dependent network structure. In real biological systems, this uncoupling is achieved through different regulatory mechanisms. Condition-dependent activation of genes is, for instance, mediated through transcriptional regulation. Upon certain environmental cues, only part of the genome is translated into an active network. Short-term environmental feedback can then be achieved by post-transcriptional or post-translational modification of this activated part of the network, whereas long-term adaptation is largely the result of selection acting at the level of the genome.

In this study, we developed a self-adaptive system, which combines a ‘bio-inspired’ artificial genome with agent-based modelling (further generally referred to as our GRN-based controller) to mimic the condition-dependent way in which only part of the genome is activated following the interaction between the robot and its environment. Using a simulated dynamically changing environment, we demonstrate that the condition-dependent activation of the GRN and its uncoupling from the genomic encoding increases the potential to evolve and adapt in a non-stable environment.

## Materials and Methods

### Implementation of the GRN based controller

The GRN-based controller actually consists of two separate layers: a bio-inspired AG and an agent-based layer. The AG is based on the model of Reil [19]. For a detailed description of the genome structure, we refer to Text S1 and Figure S1. Key to our model is the explicit distinction between signalling, regulatory and structural genes, which all have the same basic structure but differ in their ‘content region’, which specifies their functionalities. For signalling genes, the content region encodes a potential ANN structure that receives and integrates signals sensed by the robot, while for regulatory genes the content region defines the connectivity of the regulatory network, i.e. for each regulator it defines which targets the regulator can potentially interact with and the mode and extent to which the regulator can activate its targets. For structural genes, the content region defines the robot's actuators on which the structural gene can potentially act. All functions and interactions of the genes encoded in the AG are referred to as ‘potential’ because they will only become activated upon the translation of the gene into a corresponding agent (see further). The bio-inspired AG thus encodes the core GRN (the full regulatory network or entire collection of genes and all its possible interactions). The core GRN is an emergent system that changes over time by the evolutionary forces acting at the level of the genome. The total genome size consists of 10 chromosomes of 10,000 characters.

The second layer consists of an agent-based system that represents the condition-dependent instantiation of the core GRN (see Figure S2). Three types of agents have been defined, each corresponding to a specific gene type. Agents can be seen as the translation product of the genes. The agents that correspond to the gene type execute the action defined by the gene type: signalling agents include an embedded ANN, which reads the sensor input values and establishes combinations of sensor values in the (simulated) robot and channel the integrated sensor signals to the GRN by converting them into a ‘sensed value’. This ‘sensed’ value is used to activate genes in the network. Regulatory agents correspond to regulatory genes, which mediate signal transduction in the network by activating or repressing other regulatory or structural agents according to rules that are defined in the AG. A structural agent will translate the encoded information of a structural gene to an output parameter, which drives the actual actuator (e.g. wheel) of the robot. Each actuator usually receives many parameter values from different structural agents and will average these into one final value that will then be used as the control parameter (output value) for this particular actuator (see Text S2).

If a gene is translated into an agent, the ‘concentration’ of this agent depends on the expression level of the gene (which is determined by the rules encoded in the AG). In general, the higher the concentration of the agent, the higher the influence of the agent on the final output. Once translated, the concentration of the agent decays with time, mimicking protein degradation. If the concentration of the agent drops below a pre-set minimal level, the agent will be deleted. The change in concentration of an agent is determined by a default decay rate and the so-called adaptability value (AV) of the agent (see further). Adaptability values, which express the ‘added value of the agents’ presence' for the phenotype, depend on the current fitness value (see further, *Fitness function and adaptability values*). During its lifetime, the agents' concentration and survival time will increase with an enhanced adaptability value. Adaptability values of agents are thus key towards incorporating feedback from the environment (see Text S3).

### Mutational events acting at the level of the artificial genome

As evolutionary forces acting on the AG, we implemented both substitutions and duplications (see Text S4). The mutation model in our system follows the adaptive mutation model, described earlier [20]. In general, the intergenic part of the genome has a higher mutation rate than the ‘coding’ part. Also signalling genes have lower mutation rates than other genes, to guarantee that the environmental signals perceived by the robot remain relatively stable during a minimal time span. The mutation and duplication rates are gene specific and are dynamically determined by the fitness of the system. Genes with high expression levels are assumed to be under selection pressure. Therefore, the mutation rate of those genes will be lowered, mimicking the long-term effect of natural evolution in which genes that are under selection tend to be more conserved, or will be preferentially duplicated.

### Fitness function and adaptability values

In our current framework, we use the overall energy level of each simulated swarm robot as a measure of its global fitness, which is used to define the feedback from the environment to the agents and via the agents to the genes. This feedback is defined through the ‘adaptability value’. For each agent, the adaptability value (AV) is defined as a combination of the global fitness of the robot and additional values that express the dependence of the observed fitness on the specificities of a particular set of agents present in the robot at the time its fitness is evaluated. For instance, in our simulations, the adaptability value of a regulatory agent is determined by the global fitness (50%), by the overall average lifetime of the agents (assuming that an ‘agent-set’ with longer average lifetimes will have a greater long-lasting effect on the fitness) (30%), and by the number of agents active in the system, if this number ranges between 30 and 100 (20%). If the number of agents is smaller than 30, we judge the network too small to be viable. If the number of agents is larger than 100, we assume that it is hard to judge on the specificity of each of the agents. Consequently, in both cases we will decrease the contribution of the number of agents to the fitness. The details on how the AV is derived from the global fitness during the simulation can be found in Text S3).

### Simulation framework

We have used artificial life simulation [21]–[24] to see how our GRN-based simulated swarm robots perform in a changing environment. In our simulations, every robot has seven different functionalities, each of which comes with a different energy cost and energy consumption style (see Text S2, Text S5, and Table S1). The total energy consumption for one robot during one time step depends on three factors, namely 1) a basic energy consumption required for each time step, 2) the energy consumption for performing certain functionalities, and 3) extra energy consumption for aggregation, if this takes place. The robots live in a two-dimensional 90 by 90 matrix or grid in which a number of energy sources (e.g. food) are distributed. During every time step, the robots will sense the number of surrounding robots and food sources, after which the robot will determine its next action based on its input and GRN controller values. As previously stated, selection and fitness of the robots are all based on energy (in the form of food sources). Several types of food sources exist that differ from each other in the minimal amount of energy required to access the food source (see Table S2). Robots can have different energy consumption styles, each of which comes at its own cost. For instance, food sources of Type 3 (see Text S2, Text S5, and Table S2) require a minimal energy level that is higher than the maximal energy level a single robot can possess. These food sources are therefore only available to robots that have aggregated with other robots. At the same time, maintaining the aggregation with other robots will cost extra energy, but comes at the benefit of being able to acquire more costly food. Such complex functions allow robots to explore more complex behaviour [25], [26]. If a robot does not have enough energy to cover its basic living energy consumption, it will be regarded as dead and removed from the simulation. Depending on the experimental set up, different simulations were performed. The details of the simulation parameters can be found in Text S5. Note that in the simulations, the distribution of food (energy) not only depends on a random distribution function, but also on the interaction of the robots with their environment. Details of the different experiments can be found in the SI. All data are available on request.

### Related controller types

To assess the extent to which a GRN-based controller results in an improved adaptability in a dynamically changing environment, we have compared the performance of our evolutionary GRN-based controller with that of two other types of controllers. The first one is a simple controller, implemented as a static, non-evolvable ANN that transduces environmental signals over a randomly initialized network structure (referred to as a Random ANN). The second controller is an evolutionary ANN controller that uses similar genome and evolutionary operations as the one used in Bredeche et al. [27]. All control parameters, including the nodes and the weights of all edges of the ANN have been randomly initialized and the controller will respond to the environmental inputs based on these control parameters. To make the comparison between ANN and GRN controllers as fair as possible, we have limited the maximum number of agents of our GRN controller to 200, thereby reducing the inner complexity and the size of the dynamic network in our simulation. On the other hand, we also used similar feedback loop and local optimization methods for the ANN as the ones used for the GRN controller. More specifically, the ANN controller we implemented makes use of a distributed learning function that allows every edge between two nodes in the ANN to change its vector and weight value based on the feedback of the robot performance. The weights of all edges in the network structure will be optimized separately at each time step. So just as in the agent-based system, the connections and the weights of the connections between the nodes (taking the role of the agents in the GRN-based controller) in the ANN are changing dynamically in response to the environment (see Text S6). Changing the network structure thus corresponds to the genetic alteration in our bio-inspired artificial genome, whereas changing the weights of the edges corresponds to the changes we impose on the agents. This implementation therefore uses principles that are similar to the ones used by Subagdja et al. [28] and Yu et al. [29].

### Assessment of the adaptability of robot controllers in simulation experiments

The average energy level of the robot population, the average energy gain of the robot population between subsequent time steps, the number of ‘untouched’ food sources, the number of robots that survive, and the population size are all parameters used to assess the general adaptability of the robot population. The energy level reflects, for each robot, its energy at a certain time point. The average energy level then corresponds to the average of the energy levels of all robots present in the population at a certain time step (i.e. total energy of all robots divided by the population size). The energy gain between consecutive time steps reflects, for each robot separately, the net increase in energy level between the considered time points, irrespective of the historical context of the robot. The average energy gain is defined as the average of the energy increase of all robots in the population between consecutive time points (i.e. total energy gain of all robots divided by the population size).

In our set up, robots with increased adaptability will have higher energy levels, which will lead to fewer deaths and more offspring, both of which result in larger population sizes. Based on the indicators mentioned above, the average adaptability of the robots is assessed. Besides measuring the overall energy level of the robots as a measure of their adaptability, we also traced their overall phenotypic behaviour. More in particular, we assessed the evolution of the population size, and occurrences of attacks and aggregations (docking) during every time step over the whole population (not all data shown).

## Results

### Design of the bio-inspired GRN based controller

**Figure 1:**
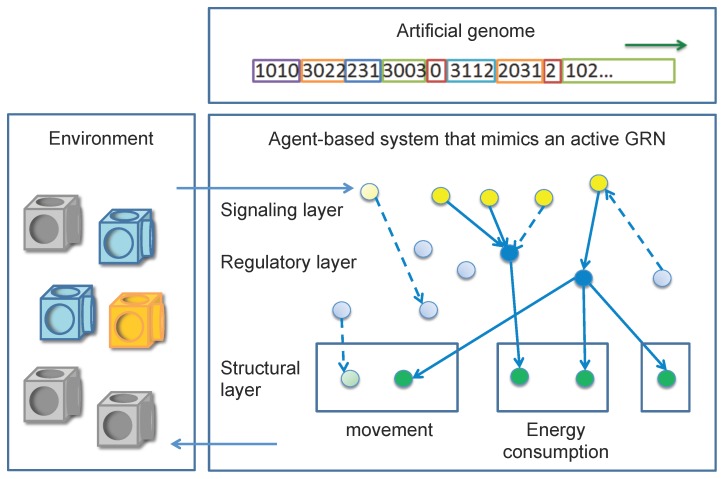
Overview of the GRN-based controller as implemented in the current study. The GRN-based controller actually consists of two separate layers. First, an artificial genome (AG) (top panel) encodes the full (core) regulatory network (lower panel, all nodes and edges), i.e. all potential interactions that can take place between signalling (yellow), regulatory (blue) and structural genes (green ‘nodes’). Evolutionary forces act at the level of this genome. Second, an agent-based layer (lower panel) that corresponds to the ‘activated’ regulatory network (colored nodes and full lines). The agent based layer mimics the translation of the core regulatory network into an activated network, following the rules embedded in the AG. Agents thus correspond to activated genes. The agent-based layer constitutes the active controller of the system and drives the behaviour of the robots (left panel). Key to our approach is the condition-dependent activation of the core genome encoded by the AG into an activated network modelled by the agent based layer resulting in the fact that only the translated part of the core network will affect the robots behaviour.

Figure 1 provides a general overview of our framework. In our approach, we assume that the genomic encoding of the cellular regulatory network and the way this encoding is translated into an activated GRN is a feature of natural systems that is key to flexible and robust adaptation. To implement the uncoupling between the genomic encoding and the part of the network that is activated in a condition-dependent way, our GRN-based controller consists of two distinct encodings of the GRN (see Materials and Methods for an extensive description). The ‘core GRN’ is encoded by the AG, which defines the genes and all their possible interactions. In this AG, genes are not pre-specified, but identified in a randomly created string of digits. Potential interactions between genes are encoded in this genome by mimicking the encoding of a transcriptional network (Text S1 and Figure S1). Although this AG-encoded GRN defines all possible interactions between genes, the set of interactions that will be activated is condition-dependent.

The condition-dependent instantiation of the core GRN is mimicked by an agent-based system. As described higher, agents can be considered as the translation products of the corresponding genes in the AG and for each gene type, a matching type of agent has been defined (Figure S2). Which part of the AG will be translated into the agent-based instantiation of the GRN depends on the encoding of the interactions in the AG: upon a certain environmental cue, a sensory agent will activate a regulatory or structural agent, according to the interaction rules that are currently present in the AG. Once this agent is activated, in turn, this agent can activate another agent following the interaction rules laid out in the AG and so on. The action of the sensory and regulatory agents thus mimics the way biological systems integrate environmental stimuli and pass them to the regulatory network. Structural agents transduce the signals perceived from the network into a pre-specified phenotypic behaviour, such as moving, docking, etc. (see Text S2).

Rather than relying on a pre-programmed static GRN defined by the AG, the GRN and its genomic encoding will evolve through the effect of evolutionary forces such as mutations and duplications. Because the long time scale over which newly evolved strains originate through mere Darwinian evolution in biological systems is very impractical in evolutionary robotics, we increased the adaptive potential of our robots by allowing for a direct feedback from the environment on the evolvability of the GRN. Agents are central in this feedback mechanism (through their adaptability value): upon increasing fitness values, agents will be able to extend their own life time (mimicking higher protein levels), allowing to directly influence the active part of the GRN. In parallel, agents will also act at the level of the genomic encoding of the GRN, e.g. by lowering the mutation rate of their respective genes, using a gene specific evolution model.

### Performance of GRN-based versus ANN-based controllers

**Figure 2:**
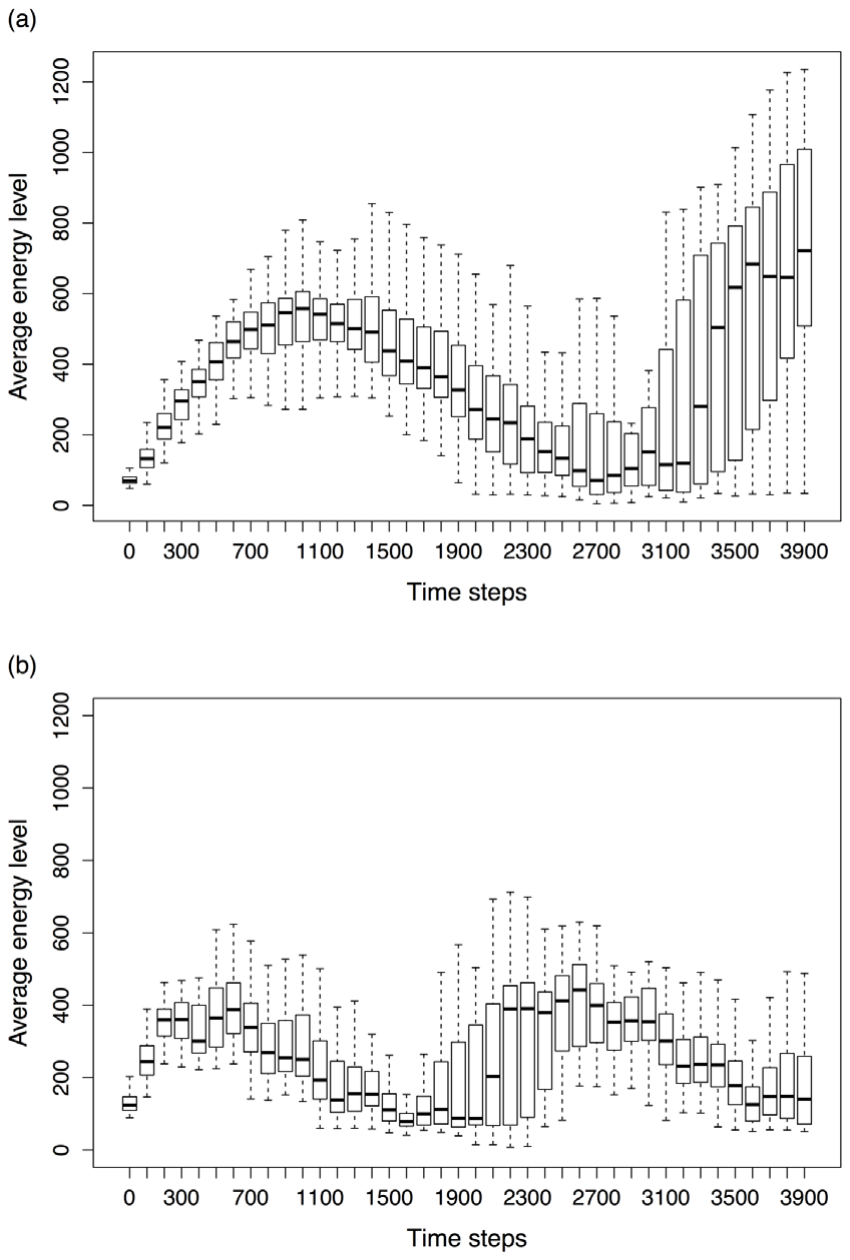
Comparison of the dynamics of the average energy level between robots with GRN (a) and ANN-based controllers (b). The x-axis represents running time measured in time steps, while the y-axis represents the populations' average energy level. The populations' average energy levels are summarized for 50 independent simulations by means of box blots in which the solid line in the box represents the median value of the average energy of all simulations, the box borders correspond to respectively the first and third quartile and the extreme values correspond to respectively the lowest and highest average energy values observed in any of the 50 simulation experiments. When the number of robots in the population drops below 100, food resources are initialised again (see Text S5).

Our GRN-based controller is different from previous controllers in several aspects. One of the most prominent features of our GRN-based controller is the uncoupling between the core and activated genome, which is achieved through the interaction between the ‘agent based layer’ and the ‘bio-inspired AG’ that defines the rules according to which the core network is translated into an activated network. To test the specific contribution of this combination to the performance of the controller, we compared with an ANN that is very similar in set up to our GRN-based controller, except for the design of its artificial genome, which does not allow for such uncoupling (see Materials and Methods). As such, we hypothesize that most of the observed differences in adaptability of robots controlled by either controller can be attributed to the differences in the design of their respective artificial genomes. We compared the performances of both controllers under a dynamically changing environment (see Materials and Methods; simulation parameters are described in Text S5 (Experiment 1) and Table S1). As a baseline, we also assessed the performance of a simple non-evolutionary ANN based controller (referred to as a random controller, see Materials and Methods). As expected, under all simulations, robots with an evolutionary controller greatly outperformed those with a random controller (not shown). The differences in adaptability, using average energy levels as indicators, between robots with evolutionary-based ANN and GRN controllers are shown in Figure 2. From these plots it is clear that, despite their similar performances at the beginning of the simulations, after a certain time, robots with a GRN based controller are more efficient in finding food sources (not shown) and therefore reach higher average energy levels than the ANN based robots. For all types of controllers, the energy levels drop after having reached an optimum for some time, which is due to food exhaustion (not shown). Interestingly, robots driven by a GRN-based controller show more variation in obtained energy levels between individuals than the ANN controller-based robots, reflecting the difference between robots with ANN and GRN-based controllers in exploring the search space and dealing with constraints imposed by the changing environment.

**Figure 3:**
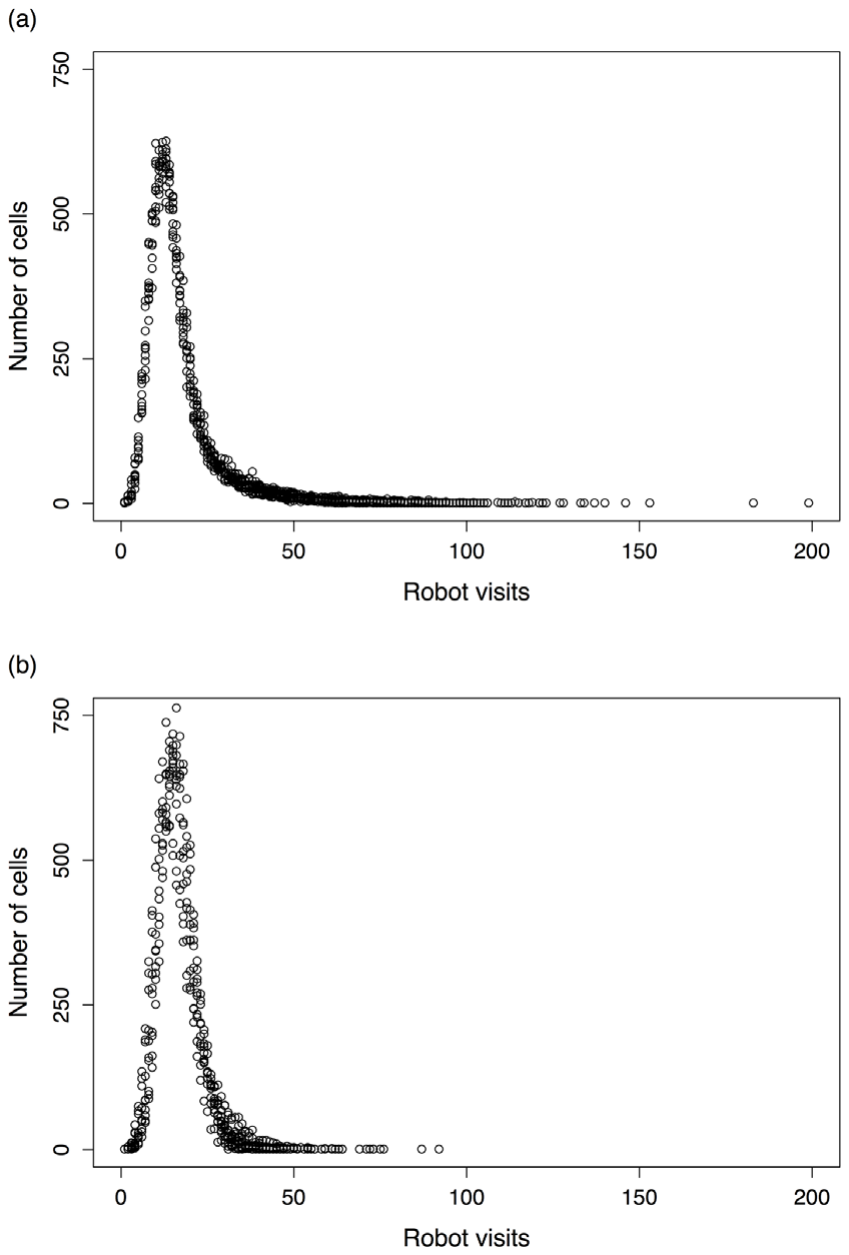
Movement behaviour for (a) ANN and (b) GRN-based robots. The X-axis represents the number of robot visits, over 50 simulations, while the Y-axis represents the number of cells that have experienced that specific number of visits (non-cumulative). Cells that have seen many visits (which is mainly true for the ANN robots) represent robots that spend much time visiting the same cell (i.e. robots have been trapped in these cells for a comparatively longer time), which implies that they do not explore the area as efficiently.

Besides measuring the overall energy level of the robots as a measure of their adaptability, we also traced their general phenotypic behaviour. For instance, Figure 3 shows the area explored by ANN and GRN robots, respectively. As can be observed, GRN robots explore the environment more evenly than ANN robots. The difference in area exploration between the two different types of robot controllers is a reflection of their more variable movement behaviour. The fact that, for ANN robots, a considerable number of cells are ‘visited’ many times (Figure 3a), implies either that, during the simulation, some robots wander around the same place for a long time or, alternatively, that more robots gather together at the same place. Considering the search for food sources and resource limitation in the environment, both situations are not ideal for the performance (adaptability) of the robots. GRN-based robots on the contrary tend to less frequently get ‘trapped’ in a certain situation (Figure 3b). They show generally more variation in the areas that get explored and therefore are less repetitive in their behaviour. This implies that GRN robots more easily change movement strategies depending on the environmental situation in which they reside.

**Figure 4:**
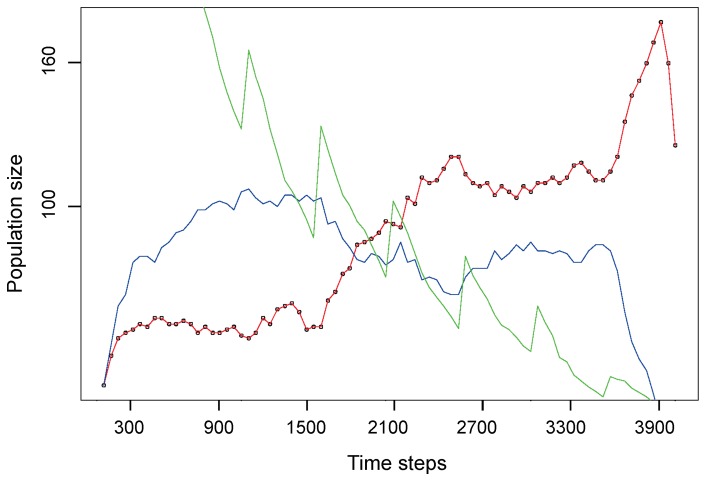
Evolution in population size of ANN and GRN-based robots in a (representative) competition experiment. The X-axis represents the different time steps during the simulation. The red curve shows the population size (Y-axis) of GRN-based robots while the blue curve shows the population size (Y-axis) of the ANN-based robots. The green curve shows the number of available food sources. Increases in the number of food sources are due to the fact that the system will add new food sources with a certain rate after a pre-set number of time steps. See text for details.

To directly compare the adaptability of our GRN controller with that of an ANN-based controller, we also performed competition experiments in which both controller types were run together in the same simulation environment (Text S5 (Experiment 2)). In this experiment, the size of the initial swarm robot population was the similar for both controller types. As can be seen in Figure 4, the population of ANN-controlled robots in general adapts faster to the initial environment than the GRN-based robot population, as is shown by the more rapid initial increase of its population size, assessed as a higher value of the first derivative of the population increase over the first 1000 time steps, a behaviour that was observed in 80% of the simulations. However, after this initial fast increase in robot population size, when food sources become more limiting and finding food more challenging, GRN-based robots tend to outcompete ANN-based robots, indicating that they can better cope with the changes in environmental conditions. Disappearance of the competitors decreases the competition imposed on the GRN-based robots, leading to a faster increase of the GRN-based population, a behaviour that was observed for all (100%) simulations, for an average running time of 4000 time steps. At the end, the rapidly increasing population causes the food resources to become exhausted, resulting again in a decrease of the GRN population.

The results of these (and other, data not shown) simulations suggest that in general the GRN-based robots gain a higher fitness and show richer phenotypic behaviour (better explore the search space, show more variable phenotypes, and are more resistant to limitations in the food resource) than ANN based robots. We hypothesize that this difference in behaviour can be mainly attributed to the uncoupling between the core and activated network which is a main feature of our GRN based controller: by mimicking the presence of condition-dependent transcriptional activation through the encoding of ‘transcriptional interactions’, an environmental condition activates only part of the ‘bio-inspired’ genome. Only this activated part of the genome will contribute to or adversely affect the robots fitness, whereas its ‘non-active’ part will randomly change (due to evolutionary operators) without directly interfering with the fitness, allowing the system to more easily escape from local optima and to explore the search space more efficiently. For the ANN-based controller on the other hand, any alteration in the network structure will cause a global influence. So once the system has reached some optimum, a small change will often have a deleterious effect, making it hard to escape from the local optimum [30].

### The ‘bio-inspired genome structure’ contributes to improved memory behavior

The specific way in which the GRN-based controller reaches its optimal energy levels reflects another important characteristic of GRN-based robots. In contrast to an ANN-based robot that re-optimizes its network each time it is subjected to a novel condition, our GRN-based system uses its bio-inspired AG to ‘store’ behaviour that was optimal under a particular environment for a sufficiently long time. When subjected to a novel environmental condition, the previous condition-specific structure might become inactivated, but remains present. This ability to store ‘good behaviour’ and to potentially disconnect it from the novel rewiring that is essential in a novel condition, allows fast re-adaptation if any of the previously observed environments is reencountered. In other words, GRN-based robots, as implemented in this study, theoretically leave a historical imprint in the system, here referred to as memory behaviour.

To further demonstrate this behaviour, we devised the following experiment in which we repeatedly imposed the same initial environmental condition and tested to what extent the GRN-based robots tend to rely or fall back on a previously evolved network to more efficiently adapt to a major switch in the environment (Text S5 (Experiment 3)). As with all simulations, food sources were restored to their initial levels as soon as the robot population drops below 100 individuals. Also here, we compared the results to those obtained with an ANN-based controller that does not make use of the ‘bio-inspired genome’ and thus should lack the memory behaviour.

**Figure 5:**
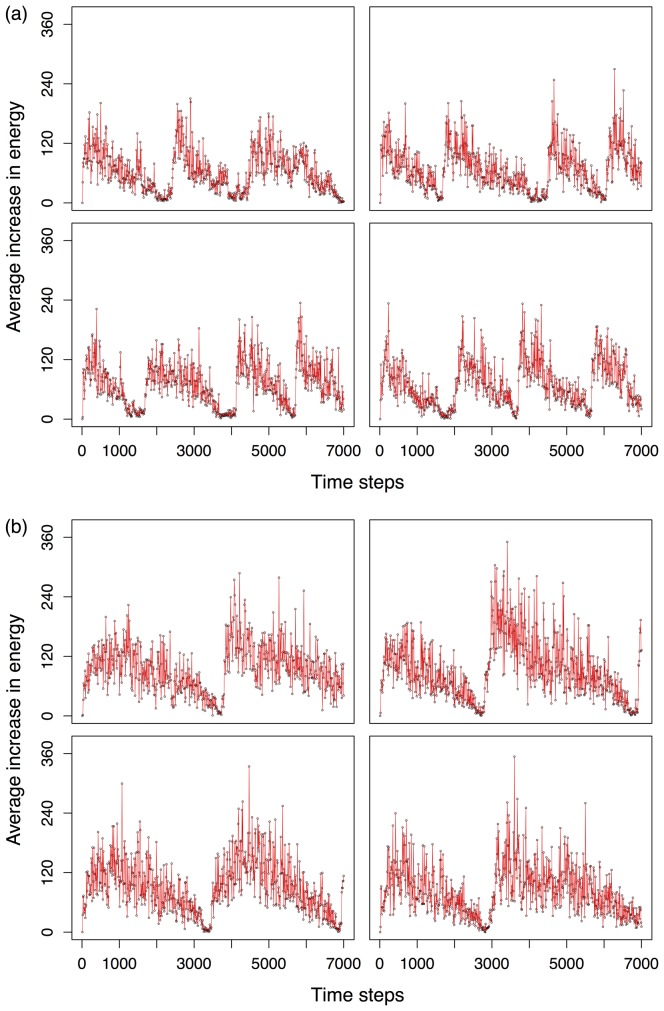
Average increase in energy for robots with ANN versus GRN controllers. The Y-axis represents the average (of the entire robot population) increase in energy measured over ten time steps, while the X-axis represent running time measured in time steps. a) Four consecutive simulations are shown for robots with ANN controllers. b) Four consecutive simulations are shown for robots with GRN controllers. Drops are caused by food resource exhaustion. When the number of robots in the population drops below 100, food resources are initialised again (see Text S5), causing the population to recover. See text for details.

Results are presented in Figure 5 and clearly show that the GRN-based controllers are more efficient than ANN-based controllers in finding food (or alternatively prey other robots), while they also survive longer, when an initial condition re-occurs, which can be inferred from the fact that the average fitness of the population (here assessed by the average increase of energy over ten time steps) is increasing despite the condition-resets. For the ANN-based controllers this behaviour is less pronounced, and sometimes even reversed. For instance, we have calculated the rate of the average energy increase from the start of the environment reset to the next environment reset. For ANN robots, the rate of the average energy increase is 12.9 energy units/10 time steps and 14,85 energy units/10 time steps for the first and second condition reset, respectively. For GRN robots, these values are 15.91 and 21.74, respectively (computed and averaged over 10 different simulations). The fact that the GRN-based robots adapt faster suggests their controller can, upon a condition reset, invoke a stored part of the GRN (or the set of agents representing the GRN) that was already previously ‘optimized’ for survival on the encountered conditions. The fact that fitness increases, suggests that the robots continue to improve an already partially optimized network structure and do not have to start evolving the network from scratch again after each condition reset.

### Disentangling the effect of the feedback from the condition dependent network-activation

Besides the condition dependent activation of the agent-driven activated network (encoded by the AG core network), feedback from the environment is also used locally and can affect individual network components (more in particular the agents' life time and the gene specific mutation rates). Although we implemented an ANN-based system that can also cope with feedback acting locally on single genes and edges and that only differs from our GRN based system in not having the condition dependent activation of the AG, we can not completely rule out that the improved performance of our GRN-based robots over the ANN-based robots can also be attributed to the differences in the way this local feedback is implemented in both systems.

Therefore, to unequivocally assess the relative impact of the way feedback is dealt with versus the conditional uncoupling of the core from the activated network, we disentangled the impact of both factors in the GRN-based system: we compared the fully functional GRN-based controller with, respectively, a GRN-based controller in which all feedback has been disabled (i.e. the feedback from the environment on the mutation rate and the agents' life time as well as the feedback responsible for the condition-dependent activation of the core genome into an agent driven activated GRN) and a GRN controller in which only the feedback from the environment on the mutation rate and the agents' life time was disabled (see Materials and Methods and Text S5).

**Figure 6:**
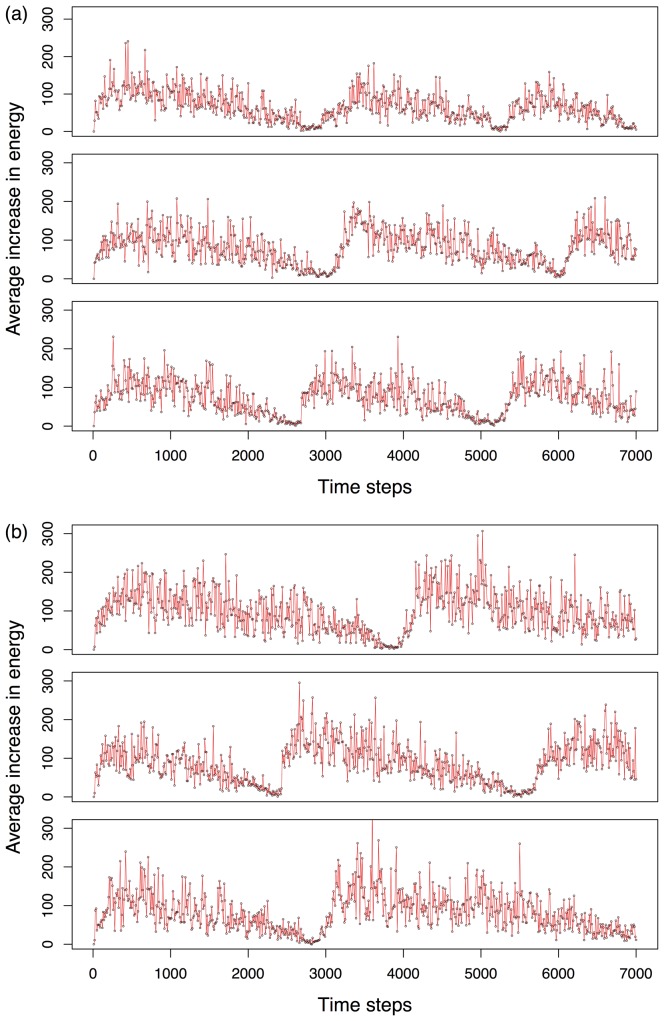
Comparison of the GRN controller robots with and without feedback. The Y-axis represents the average (of the entire robot population) increase in energy measured over ten time steps, while the X-axis represent running time measured in time steps. a) Three consecutive simulations are shown for robots with GRN controllers with all feedback disabled. b) Three consecutive simulations are shown for robots with GRN controllers with feedback enabled. Drops in average energy increase are caused by food resource exhaustion. When the number of robots in the population drops below 100, food resources are initialised again (see Text S5), causing the population to recover. See text for details.

Figure 6 shows the overall differences in adaptability of a controller where all feedback has been disabled and a controller in which all feedback has been enabled. As expected, in general, fully functional GRN-based controllers reach higher fitness, again measured as the average increase of energy over time. Although the initial performance of the robots without feedback is similar to the ones where feedback has not been disabled, the fully functional GRN robots show much better performance, particularly after the environment has been ‘reset’, suggesting that the feedback mechanisms are instrumental for the improved performance, hence adaptability, of the robots. Importantly, simulations where only the feedback from the environment on the mutation rate and the agents' life time was disabled, show a performance that is quite similar (only slightly improved) to that of fully enabled systems (data not shown), suggesting that it is indeed mainly the feedback responsible for the condition-dependent activation of the GRN that is crucial for improved adaptation.

## Discussion

The self-innovating nature or evolvability of biological systems depends on their ability to store information acquired during the past that can be reused on later occasions. For instance, bacterial systems that have been subjected to reoccurring conditions have been shown to develop memory behaviour after several rounds of training [31]. Another key factor contributing to the evolvability of biological systems is the presence of epistasis or the ability to explore a vast combination of mutations, some of which can be neutral or even deleterious to the fitness but of which the combination can largely enhance fitness values [32], [33]. Being able to explore the search space trough fitness valleys therefore is a key factor of evolving novel emergent behaviour [34]. In this work, we hypothesize that key to this memory behaviour and ability to release epistatic interactions is the decoupling of the genomic information encoding the full regulatory network (here referred to as the core GRN) from the activated part of the network. This is, amongst others, proven by the fact that cryptic variation in genomes, i.e. variations that can occur without directly interfering with the fitness, have been shown to contribute largely to the evolvability of natural systems [1], [35]. In addition, billions of years of evolution have shaped the genetic contingency of natural systems to be highly modular and degenerate. This modularity (e.g. presence of well-defined pathways) and degeneracy is the result of selecting systems that can efficiently anticipate on novel conditions without the requirement of a network rewiring that would prove detrimental in other conditions [36], [37].

Here, we tested whether imposing such bio-inspired design in which the genome and the activated part of the network are uncoupled could also improve the evolvability of an artificial self-adaptive system. To this end, we developed a robot controller that combines an artificial genome with an agent-based system that represents the activated part of the regulatory network. As in biological cells, the full regulatory network is encoded in the genome, here represented by an artificial genome consisting of both regulatory and structural genes. Depending on the environmental signals or cues, part of the encoded network is activated following the rules of transcriptional regulation. The activated part, modelled by an agent-based system, is responsible for sensing the environmental signals (signalling agents), transducing these signals through the network (regulatory agent layer, reflecting the gene products of the corresponding genes) and translating them into the proper behaviour (mediated through the structural agents). Whereas the artificial genome represents the encoding of the transcriptional network, the agents can be seen as the functional gene products (i.e. proteins) of the encoded genes. This way, the agent-based system mimics the active regulatory network and signal transduction system that is also present in naturally occurring biological systems.

Our simulations indeed show that separating the static from the conditionally active part of the network by using a bio-inspired design contributes to a better adaptive behaviour. We believe that the specific ‘memory’ behaviour and improved ability to deal with changing conditions can be mainly attributed to the ‘bio-inspired genome’ that allows uncoupling between the static and the condition-dependent part of the network. It should be noted that this work represents only a first implementation of our approach and more work is necessary to see how we can further improve on the realistic mimicking of gene regulation in artificial life forms.

## Supporting Information

Figure S1 **Fig. S1 describes the artificial genome encoding the core GRN.**
(DOCX)

Figure S2 **Fig. S2 describes the agent-based system modelling the condition dependent instantiation of the GRN encoded by the artificial genome.**
(DOCX)

Text S1 **Text S1 provides additional information on the structure of the Artificial Genome (AG).**
(DOCX)

Text S2 **Text S2 provides additional information on the robot's functionalities.**
(DOCX)

Text S3 **Text S3 provides additional information on the adaptability values.**
(DOCX)

Text S4 **Text S4 provides additional information on the mutational operators.**
(DOCX)

Text S5 **Text S5 provides additional information on the simulation parameters.**
(DOCX)

Text S6 **Text S6 provides additional information on the ANN-based controller.**
(DOCX)

Table S1 **Table S1 lists the different parameters used in the artificial life robot simulations.**
(DOCX)

Table S2 **Table S2 lists the different types of food sources used in the simulation.**
(DOCX)
