# Supplementary material for: Improving the Adaptability of Simulated Evolutionary Swarm Robots in Dynamically Changing Environments
Source: PLoS One. 2014 Mar 5;9(3):e90695. doi: 10.1371/journal.pone.0090695 (PMC3944896; doi:10.1371/journal.pone.0090695)
Supplement: Figure S1 — Fig. S1 describes the artificial genome encoding the core GRN. (DOCX) [file pone.0090695.s001.docx]

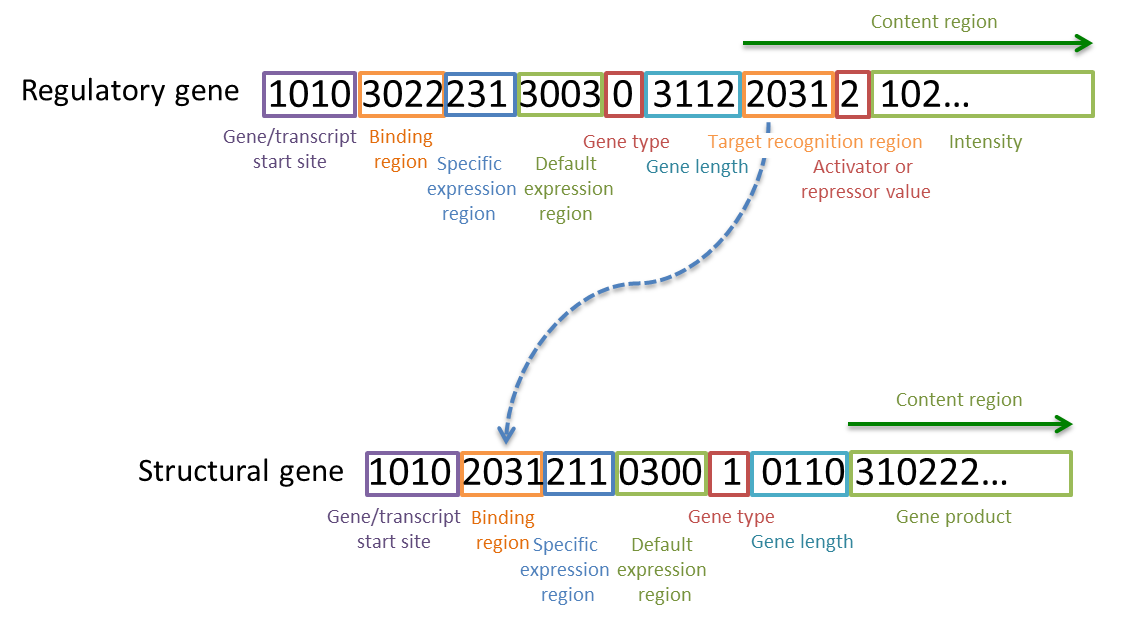


**Figure S1. Artificial genome encoding the core GRN.** Any gene, irrespective of its type, consists of the following components: a transcription start site, a gene identifier (type), a gene length region, a binding site region, an expression level region (default and gene-specific expression region), and a gene content region which is different for structural, regulatory and signalling genes.
