# Supplementary material for: Improving the Adaptability of Simulated Evolutionary Swarm Robots in Dynamically Changing Environments
Source: PLoS One. 2014 Mar 5;9(3):e90695. doi: 10.1371/journal.pone.0090695 (PMC3944896; doi:10.1371/journal.pone.0090695)
Supplement: Figure S2 — Fig. S2 describes the agent-based system modelling the condition dependent instantiation of the GRN encoded by the artificial genome. (DOCX) [file pone.0090695.s002.docx]

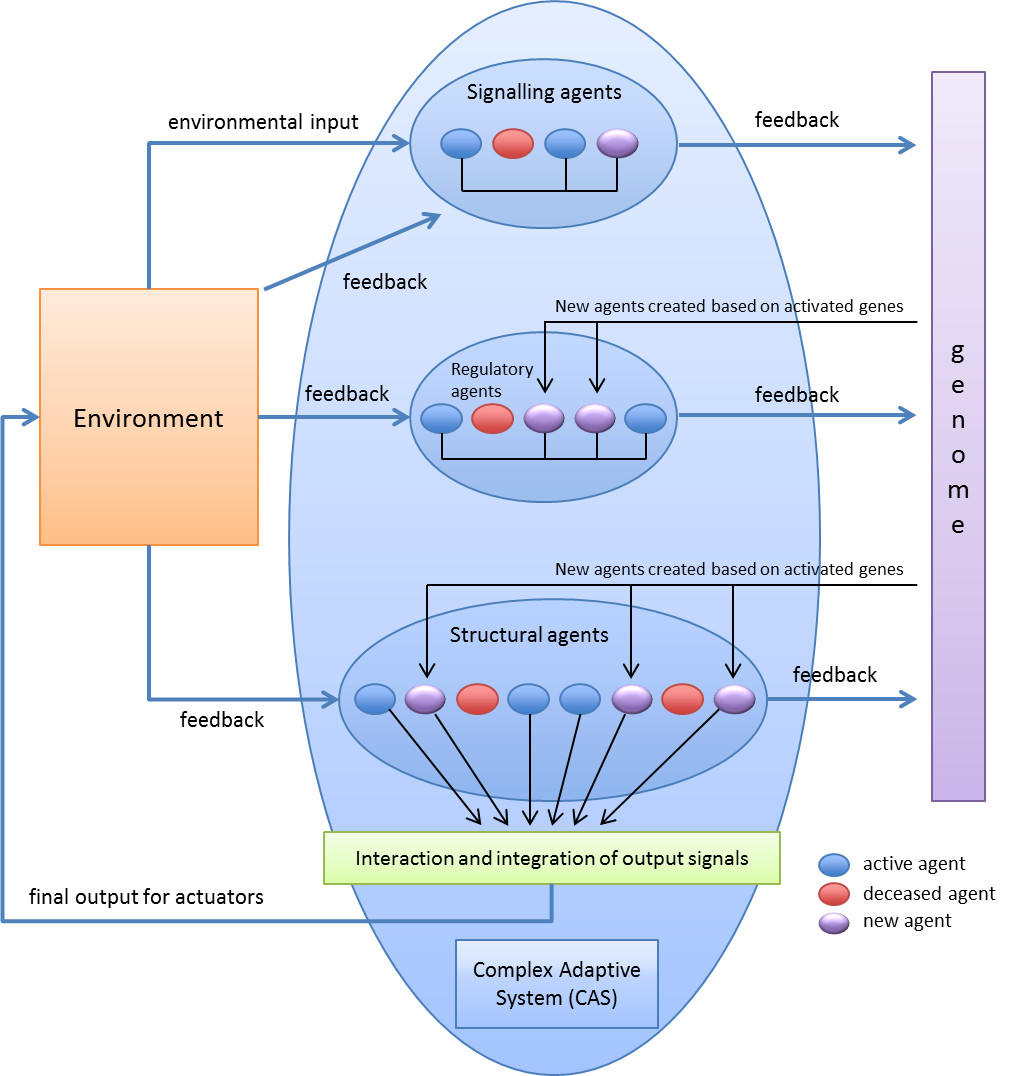


**Figure S2. Agent based system modelling the condition dependent instantiation of the GRN encoded by the artificial genome.** Three different kinds of agents are distinguished, namely signalling agents modelling the interaction between the environment and the artificial genome, regulatory agents that constitute the active part of the GRN encoded by the artificial genome, and structural agents that translate the encoded information of a structural gene to an output signal, which drives the actuators (e.g. wheel) of the robot. The agents based level is also essential in establishing the feedback from the environment to the system through the agents’ adaptability values which affect both the agents lifetime, genomic encoding, and mutations rate. See text for details.
