# Supplementary material for: Improving the Adaptability of Simulated Evolutionary Swarm Robots in Dynamically Changing Environments
Source: PLoS One. 2014 Mar 5;9(3):e90695. doi: 10.1371/journal.pone.0090695 (PMC3944896; doi:10.1371/journal.pone.0090695)
Supplement: Text S1 — provides additional information on the structure of the Artificial Genome (AG). (DOCX) [file pone.0090695.s003.docx]

**Table S1. Parameters used in the artificial life robot simulations**

| Symbol | Explanation | Default setup value |
| --- | --- | --- |
| X | horizontal position in the matrix | 90 |
| Y | vertical position in the matrix | 90 |
| MaxR | The maximum number of robots in the simulation | 200 |
| CurR | The current number of robots in the simulation | The default initial population is 100 |
| Fnum1 | The number of food sources of type 1 (see Table S2) | 500 |
| Fnum2 | The number of food sources of type 2 (see Table S2) | 100 |
| Fnum3 | The number of food sources of type 3 (see Table S2) | 50 |
| Fr | The default rate for food increase | 30% novel food sources/20 time steps |
| Re | The robot current energy level | The default initial value is 500 |
| Be | The basic energy consumption required for each time step | 7 |
| Ae | The energy consumptions for actions | based on the specific action (see Materials and Methods, main manuscript) |
| Ee | The extra energy consumption for aggregation during each time step | 1 |
| Maxe | The maximum energy for a single robot in the simulation | 1200 |
| minR | The minimal energy threshold for replication | Given by the genome of the robot. The range of this value is from 500 to 1200 |
