# Supplementary material for: Improving the Adaptability of Simulated Evolutionary Swarm Robots in Dynamically Changing Environments
Source: PLoS One. 2014 Mar 5;9(3):e90695. doi: 10.1371/journal.pone.0090695 (PMC3944896; doi:10.1371/journal.pone.0090695)
Supplement: Text S2 — provides additional information on the robot's functionalities. (DOCX) [file pone.0090695.s004.docx]

**Text S2: Robot’s functionalities**

In our simulations, each robot has seven different functionalities, each of which comes with a different energy cost and energy consumption style.

The following functionalities are performed by default and not controlled by the output of the structural genes:

1. Sensing: every time step, each robot can sense the number of other robots or food sources within a two-cell distance.
2. Preying: the robots can increase their energy level by consuming the food sources located in the same cell as the robot. For different sources of food (see Table S2), the preying might require the robot to possess different amounts of energy in advance. If preying is successful (i.e. the robot has enough energy to take the food), the food will be removed and the energy content of the food will be added to that of the robot.

The following functionalities are driven by the outputs of responsible structural genes (agents):

1. Moving: a robot can move to the surrounding cells in the two-dimensional matrix.

Energy cost: 5

Controlling outputs:

Output 1: Value > 0: move forward; Value < 0: move backward; 0 value = no movement

Output 2: Value > 0: go right; Value < 0: go left; 0 value = no movement

1. Attacking: Every time step, the robot could choose to attack another robot, which occupies the same cell. If the attack is successful, the attacking robot inherits the energy of the robot that has been attacked and the latter will be removed from the simulation.

Energy cost: 2

Controlling outputs:

Output value 3 >= 0: the robot will try to attack surrounding robots

Output value 3 < 0: the robot will not try to attack surrounding robots

1. Defending: robots can defend against an attack by another robot. This is simulated by investing a certain amount of energy (referred to as defence value). When the robot i is attacked by another robot, its defence value (def_i) plus its energy (Re_i) will be compared with the energy level (Re_h) of the attacking robot. If the attacking robot’s energy is higher (Re_h > def_i+Re_i), the defence will be broken and the defending robot’s energy will be transferred and added to that of the robot that attacks. Otherwise, the defence is successful and costs nothing. Defence values can also be accumulated during the lifespan of a robot.

Energy cost: depends on output 5 (see further)

Controlling outputs:

Output 4 decides on defending behaviour:

Value >= 0: the robot will increase the defence value

Value < 0: no increase of the defence value

Output 5 decides on how much energy is used for defending (only available when output 4 decided on defending):

1. Replication: when the energy level of robot Re_r_ exceeds a minimal threshold (minR_r_), the robot can choose to replicate. For every time step, the chance of replication (Rc) for a given robot r is based on the following equation:

Rc = $\frac{({Re}_{r}-{minR}_{r})}{500};$

(CurR<MaxR) (CurR: the current number of robots, MaxR: the maximal number of robots).

After replication, the residual energy of the replicating robot will be divided equally over the parent and daughter robots. The new robot will have the same characteristics as the parental robot. When the maximum robot population size has been reached (MaxR), the replication function will be disabled until the population has reduced again.

Energy cost: 1

Controlling outputs:

Output 6: This value determines the threshold for replication (provided the population size has not yet reached a maximal level)

1. Aggregating: At each time step, a robot can send an invitation to another robot. If the invited robot accepts the invitation, both robots will aggregate, merge their energy levels and form a new robot (referred to as a robotic organism). The GRN controllers will be integrated into the new robot by fusing their output signals. At any point, one of the joined robots can stop the aggregation and become single again. Subsequently, the separated robots will receive part of the total energy (total energy divided by the number of aggregated robots in the organism). The advantage of aggregation is that the joined ‘robotic organism’ will have more energy and greater defence capabilities. For example, the maximum energy of a robotic organism (including n robots) will be equal to $\sum_{n=1}^{n} Maxe\left( n \right)$ (Table S1). Such robotic organism will perform better on preying, defence and attacking. The disadvantage of aggregation is that, for every time step, it comes with an energy cost (see Table S1).

Energy cost: 1

Controlling outputs:

Output 7 decides whether to aggregate with another robot

Output 7 Value >= 0: the robot will aggregate with the surrounding robot value

Output 7 Value < 0: the robot will not aggregate with the surrounding robot

Output 8 Value: determines whether to disassemble (only available when the robot is part of a robotic organism):

Output 8 Value >= 0: the robot will stay aggregated

Output 8 Value < 0: the robot will disaggregate

The total energy consumption for one robot during one time step is described by the following equation (with n corresponding to the number of functionalities in time step i):

For a single robot:

Total energy consumption =$\sum_{i=1}^{n} {Ae}_{i}+Be$

For a robot that is part of a robotic organism (aggregate of robots):

Total energy consumption = $\sum_{i=1}^{n} {Ae}_{i}+Be+Ee$,

With *Ae* being the energy consumption for actions, Be being the basic energy consumption required for each time step, and Ee being the extra energy consumption for aggregation during each time step.
