# Supplementary material for: Improving the Adaptability of Simulated Evolutionary Swarm Robots in Dynamically Changing Environments
Source: PLoS One. 2014 Mar 5;9(3):e90695. doi: 10.1371/journal.pone.0090695 (PMC3944896; doi:10.1371/journal.pone.0090695)
Supplement: Text S3 — provides additional information on the adaptability values. (DOCX) [file pone.0090695.s005.docx]

**Text S3: Adaptability values**

For each agent, the adaptability value (AV) is defined by a combination of the global fitness of the robot and additional values that express the dependence of the observed fitness on the specificities of a particular set of agents present in the robot at the time its fitness is evaluated.

The adaptability value of an agent present in a robot at time step i (in case the number of agents ranges between 30 and 100):

V_i_ = ($F_{i}*50\%+A_{i}*20\%+Lf*30\%)*\frac{Ca}{Cmax}$

With Ca = the concentration level (mimicking the amount of protein product) of this agent and Cmax

With Fi being equal to the average normalized change in energy of the robot between step i and i-1: $F_{i}$ = $\frac{E_{i}-E_{i-1}}{\left( E_{i}+E_{i-1} \right)}*100$

E_i_: Energy level of the robot at step $i$ = ; E_i-1_ the energy level of the robot at the previous time step; F_i_: Fitness value of the robot at step $i$; F_i-1_ the fitness value of the robot at the previous time step

With A_i_ being equal to the number of agents being present in the robot at time step i.

With Lf being equal to the average normalized change in lifetime of the agents present in the robot between time step i and i-1:

Lf = $\frac{L_{i}-L_{i-1}}{{(L}_{i}+L_{i-1})}*100$

L_i_: average lifetime of all agents present in the robot at time step i;L_i-1_:average lifetime value of all agents present in the robot at the previous time step is

The final adaptability value at time step $i: AV=\frac{V_{i-1}+V_{i}}{2}$

With V_i_ being equal to the adaptability value at time step i and V_i-1_ being equal to the adaptability value at the previous time step.

Assuming the adaptability value of the agent is AV, the feedback effect from that agent on the gene specific expression is at a time step i: Gene specific expression (i) = $10*\frac{AV-20}{100}$ + Gene specific expression (i-1).
