# Supplementary material for: Improving the Adaptability of Simulated Evolutionary Swarm Robots in Dynamically Changing Environments
Source: PLoS One. 2014 Mar 5;9(3):e90695. doi: 10.1371/journal.pone.0090695 (PMC3944896; doi:10.1371/journal.pone.0090695)
Supplement: Text S4 — provides additional information on the mutational operators. (DOCX) [file pone.0090695.s006.docx]

**Text S4: Mutational operators**

As evolutionary forces, we implemented both substitutions and duplications. Regarding substitutions, the implementation is as follows:

In general the intergenic part of the genome has a higher mutation rate than the ‘coding’ part. The mutation rates are gene specific and are dynamically determined by the fitness of the system: non-functional sequences and genes that have not yet contributed to the individual’s fitness have a default mutation rate Nm (3*10^-5^). However, a gene with a lower contribution to the fitness function will be assigned a higher mutation rate, whereas a gene with a higher contribution has a lower mutation rate. For each gene, the current mutation rate (Gm) is dependent on both the default non-coding sequence mutation rate (Nm) and the genes’ adaptability value G1, as shown by the following equation:

$$Gm=(1-\frac{G1}{Gmax})*Nm$$

Where Gmax represents the maximum adaptability value.

These gene specific evolution models thus mimic the long-term effect of natural evolution in which genes that are under selection pressure tend to be maintained more than genes that are not.

Gene duplication is implemented as follows:

There is a software module that, at every time step, will check the expression of all genes and copies the 10 genes with the highest adaptability values. When a gene is on the list of genes with highest adaptability for more than 10 time steps, it will be regarded as a gene that qualifies to be duplicated. Like with mutations, the system also has a common background rate for duplications. Every time step, when the system searches for target genes, it also checks whether there is a gap or intergenic region between genes on the genome. If there is a gap (the minimum length being 100 bases), the program will check if there are any qualified genes with a length smaller than the gap and select this one for duplication. When all conditions have been satisfied, the candidate gene will be duplicated into the gap and removed from the list of of candidates to be duplicated (the gene might get back on the list as long as it keeps its high adaptability value). When the environmental pressure increases, most genes will receive a negative feedback and as a result, the adaptability value of their corresponding agents will decrease. As discussed previously, this will result in an increased mutation rate, which may cause destruction of the promoter region, and consequently the gene downstream of it. Fewer genes on the genome will lead to more gaps and therefore higher duplication rates. More duplicates finally will introduce more variation to the genome, with the possibility of evolving novel GRNs.
