# Supplementary material for: Improving the Adaptability of Simulated Evolutionary Swarm Robots in Dynamically Changing Environments
Source: PLoS One. 2014 Mar 5;9(3):e90695. doi: 10.1371/journal.pone.0090695 (PMC3944896; doi:10.1371/journal.pone.0090695)
Supplement: Text S5 — provides additional information on the simulation parameters. (DOCX) [file pone.0090695.s007.docx]

**Text S5: Simulation parameters**

Initially, food sources (see Table S1) are randomly distributed over the 90 x 90 grid. Each cell can only contain one food source. After a pre-set number of time steps, the system will add new food sources with a certain rate (Pr) of the same type x than those that were already present in the cell according to the following function:

Pr =$\left( \frac{500-Fnum\_x}{500} \right)*Fr$

With Fnum_x being the current total number of food sources of type x that are already present in the simulation and Fr being the default food increase rate.

In the simulation, the maximum energy level that a single robot can achieve is restricted to a value of 1200. During simulations, if the number of robots drops below 100, food sources are restored to their initial levels.

Detailed parameters that govern each simulation setup are given in the Table S1 below.

**Simulation experiments:**

Four different experimental designs were used to test different aspects of the robots. For each set up, 50 simulations were obtained.

Experiment 1: Comparing the adaptive behaviour of ANN and GRN-based robots. Here we run the simulations using the parameter setting described above for both the GRN and ANN based robots. Food sources were randomly initialized. Simulations were run for 4000 time steps. When the number of robots in the population drops below 100, food resources are initialised again. Robotic AGs were randomly initialized.

Experiment 2: Competition experiment. The simulation set up is identical as the one mentioned above except that robots with the two different controllers (ANN and GRN based) were competing in the same simulation and could mutually influence each other.

Experiment 3: Test to assess memory behaviour: The simulation set up is identical as the one mentioned for experiment 1, except that the simulation was run longer (and shown for 7000 time steps only). The experimental set up was run for several consecutive cycles in a row allowing the robots to continuously adapt their GRN. All simulations were repeated 50 times. In the main text, only few representative results are shown, as due to stochasticity, the behavior is not always exactly the same. The 50 other simulations resulted in a similar behavior, which we assessed as follows:

1. the difference between the maximal energy level between the last and the first cycle as a measure of the global energy gain and averaging those figures over the 50 simulations.
2. by calculating for how many simulations the energy is monotonically increasing over the different cycles (no fall-backs)
3. by calculating the average energy increase between two consecutive cycles (also assessed at the point where in each cycle the maximal energy level is obtained)

Experiment 4: Set up was identical as the one in experiment 3 except that here we compared the performance of:

- the GRN-based controller with full condition feed back (that is feedback of the environment on the life time of the agents, the gene specific mutation rate, and the condition dependent activation of the AG in activated agents
- the GRN-based controller with reduced feedback i.e. the feedback from the environment on the life time of the agents and the gene specific mutation rate (through the AV values) is disabled, but these robots can still uncouple the core network encoded by the AG from the condition-dependent activated network (data not shown)
- All input from the environment was disabled

All simulations were repeated 50 times. In the main text, only few representative results are shown, because, due to stochasticity the behavior is not always exactly the same. The 50 other simulations resulted in a similar behavior, which we assessed as in experiment 3 (see higher).
