# Supplementary material for: Improving the Adaptability of Simulated Evolutionary Swarm Robots in Dynamically Changing Environments
Source: PLoS One. 2014 Mar 5;9(3):e90695. doi: 10.1371/journal.pone.0090695 (PMC3944896; doi:10.1371/journal.pone.0090695)
Supplement: Text S6 — provides additional information on the ANN-based controller. (DOCX) [file pone.0090695.s008.docx]

**Text S6: The ANN-based controller**

In the ANN controller, each edge corresponds to an agent that responds to the global fitness of the robot’s Fi in the following way (as determined by its adaptability value)

The adaptability value of the agent present in the ANN at time step i is:

$$\mathrm{AV}_{i}=\frac{F_{i-1}+F_{i}}{2}$$

Two parameters (AV_max_ and AV_change_) will be assigned to each agent. These two parameters can differ amongst different agents. If AV_i_ is smaller than AV_max_, the agent will calculate the value AV_distance_ at time step i:

AV_distance(i)_= (AV_max_-AVi)+ AV_distance(i-1)_

If AV_distance(i)_ is greater than AV_change_, the agent will change the weight parameter at that time step otherwise the agent will keep the same weight parameter. If AV is greater than AV_max_ , AV_distance_ will become 0.

If the agent decides to change the weight parameter (W), it will add or subtract a certain value, based on AV_distance_. The value increase C at time step i is determined as:

C = AV_distance_ *R_change_

Where R_change_ is randomly assigned in the range of 0% to 50%, decided by the gene.

The mutation rate of the genome is based on the energy level of the robot. The lower the energy level, the higher the mutation rate. The mutation rates ranges from 2*10^-4^ to 0 (for the 350 genes in genome).
