# Supplementary material for: Improving the Adaptability of Simulated Evolutionary Swarm Robots in Dynamically Changing Environments
Source: PLoS One. 2014 Mar 5;9(3):e90695. doi: 10.1371/journal.pone.0090695 (PMC3944896; doi:10.1371/journal.pone.0090695)
Supplement: Table S2 — lists the different types of food sources used in the simulation. (DOCX) [file pone.0090695.s010.docx]

**Table S2: Different types of food sources used in the simulation**

| Food type | Food energy | Distribution range (by coordinate value) | Requirement |
| --- | --- | --- | --- |
| Type 1 | 300 | X:0-60;Y:0-90 | No requirement |
| Type 2 | 1000 | X:61-80;Y:0-90 | Can only be eaten by robots that have energy levels >= 800 |
| Type 3 | 3000 | X:81-90;Y:0-90 | Can only be eaten by robots that have energy levels >= 2000 |
